# Epidural analgesia information sessions provided by anesthetic nurses: impact on satisfaction and anxiety of parturient women a prospective sequential study

**DOI:** 10.1186/s12871-022-01647-z

**Published:** 2022-04-12

**Authors:** Quentin Cherel, Julien Burey, Julien Rousset, Anne Picard, Dimitra Mirza, Christina Dias, Hélène Jacquet, Paule Mariani, Nathalie Raffegeau, Isabelle Saupin, Marie Bornes, Nathanaël Lapidus, Christophe Quesnel, Marc Garnier

**Affiliations:** 1 Service d’anesthésie, réanimation et médecine périopératoire, Hôpital Tenon, Sorbonne Université, GRC29, APHP, DMU DREAM, 4 rue de la Chine, 75020 Paris, France; 2 Service de gynécologie-obstétrique, Hôpital Tenon, Sorbonne Université, APHP, DMU ORIGYNE, 4 rue de la Chine, 75020 Paris, France; 3 Département de Santé Publique, Hôpital Saint-Antoine, Sorbonne Université, INSERM, Institut Pierre Louis d’Epidémiologie et de Santé Publique IPLESP, APHP, Sorbonne Université, 184 rue du Faubourg St Antoine, 75012 Paris, France

**Keywords:** Epidural analgesia, Information, Anesthetic nurse, Satisfaction, Anxiety

## Abstract

**Background:**

Information on epidural analgesia delivered to parturient women is frequently incomplete, making it difficult for expectant mothers to make an appropriate choice for their delivery. We assessed the impact of a multimodal information session on epidural analgesia delegated to anesthetic nurses on new-mothers’ satisfaction.

**Methods:**

We performed a prospective sequential study including parturient women who gave birth with epidural analgesia. During the first period, information on epidural analgesia was delivered by anesthetists during the scheduled anesthesia consultation, according to French standard-of-care. Then, a dedicated information session about epidural analgesia provided by anesthetic nurses was implemented. The primary endpoint was the satisfaction of women with the quality of information received. Main secondary endpoints were knowledge of women about epidural analgesia, anxiety before epidural catheter placement, and satisfaction with delivery.

**Results:**

259 and 298 women were included during the first and second periods respectively, among whom 178 and 188 were analyzed. Information on epidural analgesia delivered by anesthetic nurses was associated with improvement of new-mothers’ satisfaction with information received (9 (8–10) vs. 10 (9–10) – *p* < 0.001). Moreover, information delivered by anesthetic nurses was associated with decreased anxiety before epidural catheter placement (4 (1–8) vs. 3 (1–6) – *p* = 0.006) and increased satisfaction with delivery (8 (7–10) vs. 9 (8–10) – *p* = 0.01). Women’s knowledge on epidural analgesia was durably increased when information was delivered by anesthetic nurses compared to conventional information by anesthetists. After adjustment, the only variable associated with both new mothers’ satisfaction with information and delivery was the information session taught by anesthetic nurses.

**Conclusions:**

Information sessions on epidural analgesia delivered by anesthetic nurses was associated with improved satisfaction of women with their delivery. Such information sessions may be used in maternity wards to improve new-mothers’ childbirth experience.

**Supplementary Information:**

The online version contains supplementary material available at 10.1186/s12871-022-01647-z.

## Introduction

Epidural analgesia was introduced to decrease the pain associated with labor in the late 1940’s [1]. Indeed, for most women labor causes severe pain, which affects the expectant mother, father and child. Labor-related pain induces neuropsychological disorders in parturient women and may increase incidence of post-partum depression and post-traumatic stress disorder [2]. Severe labor pain may also impact the level of anxiety and stress felt by future fathers, and their support to women during delivery [3]. Thus, in the absence of contraindications, maternal request is a sufficient medical indication for pain relief during labor [4]. For this purpose, epidural analgesia offers many advantages over systemic analgesia, in particular over systemic opioids [5]. However, several maternal side-effects associated with epidural analgesia have been reported such as hypotension, urinary retention, delayed second stage of labor and risk of dural puncture and subsequent headache [6]. Epidural analgesia has also been associated with more frequent category II and III fetal heart rate tracing during labor and lower umbilical cord blood pH at birth, even if the incidence of severe child adverse outcomes is similar to that observed in the absence of epidural analgesia [7, 8]. It is therefore the responsibility of the anesthetic staff to correctly inform parturient women about advantages and proven side effects of epidural analgesia, so that expectant mothers can make an appropriate choice for their delivery. This is one of the main goals of the anesthesia consultation, which must take place during the third trimester of pregnancy according to French health authorities’ recommendations [9]. However, several constraints may impact the quality of this information, in particular the relatively short time dedicated to the anesthesia consultation before delivery that predominantly targets healthy young women with an ASA 1 class. Consequently, practical information on epidural analgesia remains frequently incomplete, notably in terms of explanations about risks [10, 11].

The main aims of this study were to assess the impact of a dedicated information session on epidural analgesia delegated to anesthetic nurses on 1) new mothers’ satisfaction with information received at hospital about epidural analgesia, 2) acquired knowledge about epidural analgesia assessed by an objective test, and 3) global satisfaction with their childbirth.

## Methods

This manuscript adheres to the STROBE guidelines. STROBE checklist is provided in the online additional material (Additional file 1).

### Patient population

All parturient women over 18 years were screened for eligibility before their anesthesia consultation, scheduled during the 8^th^ month of pregnancy according to French health authorities recommendations [9]. Then, all women who did not meet non-inclusion criteria were included.

#### Exclusion criteria

Parturient women whose understanding of French language was insufficient to be correctly informed were not included, as well as women who declined to participate. Parturient women who had premature delivery before their scheduled anesthesia consultation and thus received information during the emergency anesthesia consultation just before their delivery were not included. In addition, women with planned caesarean section were not included, as they would not have epidural analgesia. Finally, women who gave birth without epidural analgesia were excluded from the final analysis.

### Conduct of the study

This study is a monocentric prospective sequential study, performed at Tenon University Hospital, Paris, France, between February and September 2019. During the first period (February-April 2019), parturient women were informed about epidural analgesia by the anesthetist during the planned anesthesia consultation. In our center, this information is based on standardized oral information focusing on 3 points: benefits of epidural analgesia for vaginal delivery and in case of emergency cesarean section, main side-effects of epidural analgesia during placement and use, and how and when the epidural catheter will be removed in the postpartum period.

A dedicated collective information session on epidural analgesia organized into groups of 6 women and given by anesthetic nurses just before the anesthesia consultation was then implemented in our establishment on 9 May 2019. This information session was designed by the anesthetic staff, based on a preliminary assessment of content preferences collected from 50 parturient women just after their anesthesia consultation on January 2019. In its final version, this free 40-min information session included a digital presentation covering the same 3 aspects previously explained by the anesthetists, followed by a discussion with the participants and a presentation of the equipment used by the anesthetist for epidural catheter placement and by parturient women for patient-controlled epidural analgesia. Thus, during the second study period, information on epidural analgesia was delivered collectively by anesthetic nurses, while discussions on the indications, contraindications and choice of expectant mothers were still conducted individually by the anesthetist according to French law.

### Measurements and data handling

A knowledge test, made of 4 multiple-choice questions investigating four different fields related to epidural analgesia (Additional file 2), was performed on all parturient women: 1) just before the anesthesia consultation (first period) or collective information session (second period); 2) just after the anesthesia consultation; and 3) just before epidural catheter placement the day women came to the hospital for their delivery. Correct answers were not given to women, so that there was no learning and score progression attributable to taking the test. The rating for each question, made of 5 propositions, was quoted according to the principle of discordance (no discordance: 1 point; 1 discordance: 0.5 point; 2 discordances: 0.2 point; ≥ 3 discordances: 0 point). Then, ratings for the 4 questions were added up to obtain a global knowledge score on epidural analgesia on 4 points for each woman, at each time-point. The knowledge test was validated prior to its use in the study, on 50 pregnant women before and just after their anesthesia consultation on January 2019 to ensure that propositions were clear and appropriately understood, and that it was sensitive to detecting an improvement in knowledge.

In addition, satisfaction of the anesthetic physicians with the course of the anesthesia consultation was collected just after the consultation.

Then, the day after they gave birth, new mothers were asked to quote using a 10-level numeric scale: 1) satisfaction with the information received at the hospital during their pregnancy regarding epidural analgesia; 2) satisfaction with epidural analgesia efficacy; 3) global satisfaction with their delivery; and 4) anxiety felt before epidural catheter placement.

General demographic data (age, number of previous pregnancies and uses of epidural analgesia, current profession) and data on the current pregnancy (whether the parturient took childbirth preparation classes, searched for other information sources, etc.) and delivery (complicated vaginal delivery, conversion to caesarean section, etc.) were also collected.

### Primary and secondary endpoints

The primary endpoint was the satisfaction of new mothers with information received at hospital about epidural analgesia, collected on the first day post-partum.

Secondary endpoints were: global satisfaction with childbirth, satisfaction with epidural analgesia efficacy, anxiety felt before epidural catheter placement, knowledge of parturient women about epidural analgesia, and satisfaction of anesthetists with the anesthesia consultation course.

### Sample size calculation

A sample size calculation was made prior to the beginning of the study. During the preliminary phase of the study in January 2019 (assessment of the preferences on the content of an extended information session and validation of the knowledge test on 50 parturient women), satisfaction with the information received about epidural analgesia in the standard-of-care including information entirely given by the anesthetist was rated at 8/10 ± 3/10. Then, 190 parturient women in each group were required to show a difference of ± 1 point with the dedicated information session delegated to anesthetic nurses, with α = 5% and power (1-β) = 90%.

### Statistical analyses

Distributions are reported as median (25^th^-75^th^ percentile) for continuous variables and counts (proportions) for qualitative ones. Distributions were compared between the two periods using Mann–Whitney-Wilcoxon tests, and chi-square or Fisher exact tests – as appropriate – for continuous and qualitative variables, respectively. To identify factors associated with satisfaction on information received and global satisfaction with delivery, all covariates deemed clinically relevant were included in a multivariable linear regression model. All tests were two-tailed and *p* values below 0.05 were considered significant. Statistical analysis was performed using GraphPad Prism 8.4.3 (San Diego, CA, USA) and R version 4.0.1 (R Foundation for Statistical Computing, Vienna, Austria).

## Results

### Inclusions and women’s characteristics

**Fig. 1:**
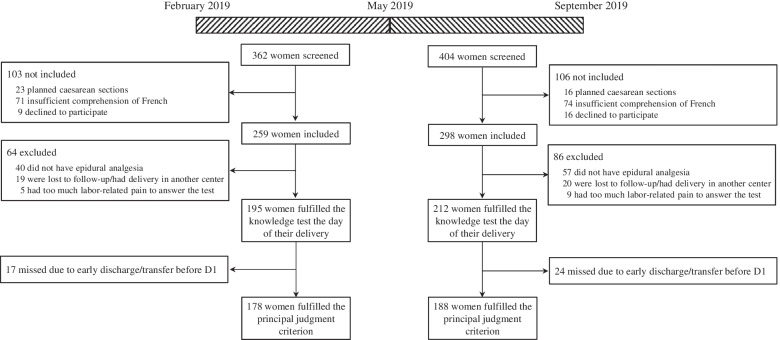
Flow chart of the study

During the first and second periods, 362 and 404 women were screened, of whom 259 and 298 were included before the anesthesia consultation, 195 and 212 fulfilled the knowledge test on the day of their delivery, and 178 and 188 had complete data about the main judgment criterion and were finally analyzed (Fig. 1). The main reasons for exclusion were the absence of epidural catheter placement and lost to follow-up due to delivery in another center (Fig. 1).

Demographic characteristics of parturient women are presented in Table 1. Median ages of women were 31.6 (29.0–35.0) and 32.2 (28.1–35.2) years during the first and second periods, respectively. Eighty-six (48%) and 88 (47%) were multiparous, among which 72 (84%) and 79 (90%) had previous epidural analgesia and were mainly satisfied with it. The main socio-professional categories represented were non-workers, employees and middle-class professions with no difference between groups. Nearly half of women took childbirth preparation classes, which are taught by self-employed midwives during the second trimester of pregnancy for women who want it, with the aim to prepare expectant mothers for delivery, in French healthcare organization.

**Table 1:** Characteristics of parturient women included in the study

	**Period 1**	**Period 2**	***P* value**
	*n* = *178*	*n* = *188*	
Age, *years*	31.6 (29.0–35.0)	32.2 (28.1–35.2)	0.65
Gravida	2 (1–2)	1 (1–2)	0.21
Parity	0 (0–1)	0 (0–1)	0.83
Multipara, *n (%)*	86 (48%)	88 (47%)	0.83
Previous epidural analgesia in multiparous women, *n (%)*	72 (84%)	79 (90%)	0.27
Satisfaction of previous epidural analgesia, *yes/no*	61/11	61/18	0.30
Socio-professional grade, *n (%)*			
Non-working	44 (25%)	56 (30%)	
Students	9 (5%)	8 (4%)	
Workers	0 (0%)	0 (0%)	
Employees	49 (27%)	50 (27%)	0.92
Craftsman, merchant, self-employed	12 (7%)	11 (6%)	
Middle class, intermediate profession	41 (23%)	38 (20%)	
Upper Class	23 (13%)	25 (13%)	
Childbirth preparation classes following, *n (%)*	86 (48%)	84 (45%)	0.53
Pre-eclampsia	7 (4%)	4 (2%)	0.37
Twin pregnancy	2 (1%)	0 (0%)	0.24
Preterm birth	4 (2%)	4 (2%)	0.99
Complicated vaginal delivery			
Instrumental vaginal delivery	26 (15%)	28 (15%)	0.99
Episiotomy	26 (15%)	16 (9%)	0.07
Perineal tear	74 (42%)	102 (54%)	0.02
Manual uterine exploration	16 (9%)	19 (10%)	0.73
Conversion to caesarean section	37 (21%)	30 (16%)	0.28
Post-partum hemorrhage			
Immediate	8 (4%)	10 (5%)	0.83
Delayed	2 (1%)	2 (1%)	
Fetal distress during delivery			
Fetal heart rate abnormalities	35 (20%)	41 (22%)	0.70
Meconium-stained amniotic fluid	23 (13%)	22 (12%)	0.75
New-born resuscitation	0 (0%)	0 (0%)	-

Continuous variables are reported as median (25^th^-75^th^ percentile). Distributions were compared between the two periods with the chi-square or Fisher exact test, as appropriate, for qualitative variables and with the Mann–Whitney-Wilcoxon test for continuous variables

### Knowledge of parturient women about epidural analgesia

**Fig. 2:**
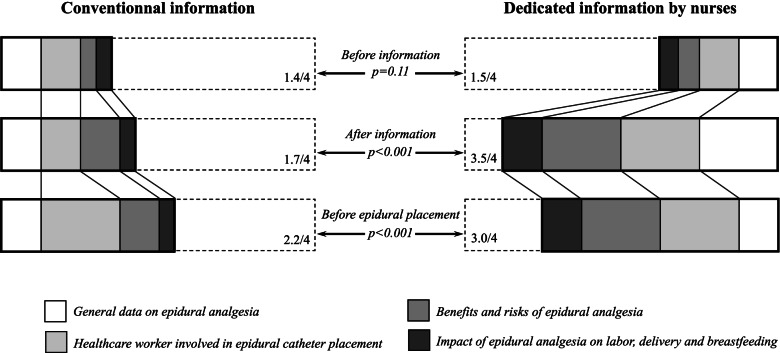
Progression of knowledge of parturient women on epidural analgesia either informed by the anesthetist during the anesthesia consultation (*left*) or anesthetic nurses during the dedicated information session (*right*), from before any information delivered at the hospital (*upper line*), to after information has been delivered (*middle line*) and before epidural catheter placement on the day of delivery (*lower line*)

Median knowledge scores before information, either delivered by anesthetists during the first period or anesthetic nurses during the second period, were low and similar between the 2 groups (1.4 (0.9–2.1) and 1.5 (1.0–2.2) – *p* = 0.11) (Fig. 2). Information delivered either during the anesthesia consultation, or the collective session animated by nurses, both significantly increased immediate knowledge of parturient women on epidural analgesia (+ 0.3 (-0.2- + 0.8) point and + 1.5 (+ 0.8- + 2.3) points, respectively – Table 2). However, knowledge was much improved by the dedicated information delivered by anesthetic nurses (3.5 (2.7–4.0) vs. 1.7 (1.2–2.5) – *p* < 0.001) (Fig. 2 and Table 2). Results of the knowledge test slightly decreased several weeks later on the day of delivery during the second period, but persistent level of knowledge remained better following the information delivered by anesthetic nurses (3.0 (2.2–3.5) vs. 2.2 (1.5–2.5) – *p* < 0.001) (Table 2). Consultation of sources of information other than that received at the hospital was halved between the first and second period (22.5% vs. 11.7%—*p* = 0.008) (Table 2).

**Table 2:** Results on main and secondary judgment criteria

	**Period 1**	**Period 2**	***P* value**
	*n* = *178*	*n* = *188*	
Duration of the anesthetic consultation, *min*^a^	10 (8–12)	8 (6–10)	< 0.001
Satisfaction of the anesthetist on the consultation, *10-level numeric scale*^a^	7 (6–8)	8 (7–9)	< 0.001
Knowledge test on epidural analgesia result, *rating on 4 points*^b^			
Before any information	1.4 (0.9–2.1)	1.5 (1.0–2.2)	0.11
After the anesthetic consultation	1.7 (1.2–2.5) ^#^	3.5 (2.7–4.0) ^#^	< 0.001
Before epidural catheter placement	2.2 (1.5–2.5) ^§^	3.0 (2.2–3.5) ^§^	< 0.001
Knowledge test on epidural analgesia result, *individual changes*^b^			
Between before and after the anesthetic consultation	+ 0.3 (-0.2 – + 0.8)	+ 1.5 (+ 0.8 – + 2.3)	< 0.001
Between after the anesthetic consultation and before epidural catheter placement	+ 0.3 (-0.3 – + 0.8)	-0.4 (-1 – 0)	< 0.001
Satisfaction of new mothers, *10-level numeric scale*			
Satisfaction on the information received about epidural analgesia	9 (8–10)	10 (9–10)	< 0.001
Satisfaction on epidural analgesia efficacy	10 (8–10)	10 (8–10)	0.21
Global satisfaction on delivery	8 (7–10)	9 (8–10)	0.01
Consultation of at least one other information source on epidural analgesia after the anesthetic consultation, *yes/no*	40/138	22/166	0.008
Internet	29	14	
Book, written support	5	3	0.10
Forum, oral discussions	9	5	
Anxiety felt just before epidural catheter placement, *10-level numeric scale*	4 (1–8)	3 (1–6)	0.006

^a^ Data available for 168 among the 178 women of the first period and 188 women of the second period

^b^ Test results were obtained at the 3 time-points for 195 and 212 women during the first and second periods, respectively (cf. Figure 1)

^#^*p* < 0.05 for comparison with the rating before any information using the signed-rank Wilcoxon test

^§^ *p* < 0.05 for comparison with the rating after the anesthetic consultation using the signed-rank Wilcoxon test

### Women satisfaction

The information delivered by anesthetic nurses was associated with higher satisfaction with the information about epidural analgesia received at the hospital (9 (8–10) vs. 10 (9–10) – *p* < 0.001). In addition, global satisfaction of women with their delivery was also increased during the second period (8 (7–10) vs. 9 (8–10) – *p* = 0.01). Satisfaction with epidural analgesia efficacy was identical between the 2 periods (10 (8–10) vs. 10 (8–10) – *p* = 0.21). Finally, the level of anxiety felt by parturient women just before epidural catheter placement was decreased during the second period compared to the first (4 (1–8) vs. 3 (1–6) – *p* = 0.006).

### Anesthetic point-of-view

After the implementation of the information session given by anesthetic nurses, duration of the anesthesia consultation significantly decreased from 10 (8–12) to 8 (6–10) minutes (*p* < 0.001). At the same time, global satisfaction of anesthetists with the consultation course increased from 7 (6–8) to 8 (7–9) (*p* < 0.001) (Table 2).

### Factors associated with new mothers’ satisfaction

After adjustment, the only variable positively associated with new mothers’ satisfaction with information about epidural analgesia was information delivered by anesthetic nurses (+ 0.76 (0.48–1.04) point – *p* < 0.001), while increasing age was negatively associated with satisfaction with information received (-0.04 (-0.07 – -0.01) point per one-year increase – *p* = 0.005) (Table 3). Accordingly, information delivered by anesthetic nurses was the only variable positively associated with global satisfaction with delivery (+ 0.5 (0.2–0.8) point – *p* = 0.004), while the occurrence of a post-partum hemorrhage was negatively associated with this outcome (-1.0 (-1.7 – -0.2) point – *p* = 0.01) (Table 4). The other variables, notably including age, multipara, socio-professional grade or childbirth preparation classes, were not associated with satisfaction of new mothers with either information received or childbirth experience (Tables 3 and 4).

**Table 3:** Multivariable analysis of factors associated with new mothers’ satisfaction with information received at the hospital on epidural analgesia

	**Linear regression coefficient**	**95% confidence interval**	***P* value**
Age^a^	-0.04	(-0.07 – -0.01)	0.005
Multipara	-0.20	(-0.43 – 0.03)	0.09
Epidural analgesia use for previous delivery	0.02	(-0.29 – 0.33)	0.89
Socio-professional grade^b^			
Non-working (*reference*)		(reference)	
Employees	-0.22	(-0.61 – 0.16)	0.25
Craftsman, merchant, self-employed	-0.34	(-0.95 – 0.27)	0.28
Middle class, intermediate profession	-0.35	(-0.77 – 0.06)	0.09
Upper Class	-0.19	(-0.70 – 0.31)	0.45
Students	-0.08	(-0.76 – 0.61)	0.83
Duration of the anesthetic consultation^c^	0.01	(-0.01 – 0.03)	0.41
Childbirth preparation classes	-0.24	(-0.58 – 0.09)	0.15
Information on epidural analgesia by anesthetic nurses	0.76	(0.48 – 1.04)	< 0.001

^a^ Per one-year increase

^b^ Compared to non-working women

^c^ Per one-minute increase

**Table 4:** Multivariable analysis of factors associated with new mothers’ satisfaction with their delivery

	**Linear regression coefficient**	**95% confidence interval**	***P* value**
Age	-0.02^a^	(-0.06 – 0.01)	0.25
Multipara	0.04	(-0.38 – 0.46)	0.86
Socio-professional grade^b^			
Non-working (*reference*)	0	(reference)	
Employees	-0.15	(-0.61 – 0.31)	0.51
Craftsman, merchant, self-employed	0.11	(-0.65 – 0.88)	0.77
Middle class, intermediate profession	-0.15	(-0.65 – 0.36)	0.57
Upper Class	0.13	(-0.47 – 0.73)	0.67
Students	-0.21	(-1.08 – 0.67)	0.64
Childbirth preparation classes	-0.11	(-0.53 – 0.30)	0.58
Information on epidural analgesia by anesthetic nurses	0.50	(0.16 – 0.84)	0.004
Pre-eclampsia	0.54	(-0.47 – 1.55)	0.29
Preterm birth	0.14	(-1.03 – 1.31)	0.82
Complicated vaginal delivery	-0.16	(-0.56 – 0.25)	0.45
Conversion to caesarean section	-0.49	(-1.04 – 0.05)	0.09
Post-partum hemorrhage	-0.99	(-1.67 – -0.23)	0.01
Fetal distress during delivery	-0.10	(-0.48 – 0.27)	0.58

^a^ Per one-year increase

^b^ Compared to non-working women

## Discussion

Our main results may be summarized as follows: a session dedicated to information on epidural analgesia given by anesthetic nurses was associated with 1) improved satisfaction of new mothers with information received on epidural analgesia; 2) better immediate and medium-term knowledge about epidural analgesia; 3) decreased anxiety related to epidural catheter placement; 4) improved satisfaction of new mothers with their delivery.

Complete and objective information is mandatory so that patients can make appropriate choices regarding their health and become truly active players in their medical care. Anesthetic practice, and notably epidural analgesia for labor, seems to be particularly concerned by this ethical and regulatory obligation. Extensive information is a strong wish of parturient women [12], which should cover the technical aspect and benefits and risks of epidural analgesia, in addition to all the other usual tasks of anesthetic consultation regarding the collection of patients' histories, treatments, etc. This objective may seem incompatible with daily practice, in which the time devoted to a consultation is generally insufficient to fully accomplish all these tasks. Consequently, anesthetists mainly deliver information summarized on the points they consider important [13], sometimes in the form of a pre-established monologue, which can be a source of frustration for both anesthetists and expectant mothers, and may be quickly forgotten [14].

Previous teams have implemented alternative methods to inform about epidural analgesia. Written information, notably in the form of illustrated booklets has been associated with improvement of women’s knowledge [15, 16]. Despite their practicality and ease of implementation, written brochures have important limitations, such as not being adapted to the pre-existing individual knowledge and expectations of patients, or of being poorly read [17]. Others have assessed the impact of videos, with mitigated results on satisfaction and anxiety [18–21]. More recently, numerous websites have emerged, providing very accessible information, but often of very imperfect quality and relevance [22]. Thus, parturient women often have confused and preconceived ideas about epidural analgesia, which may cause inappropriate choices such as delaying epidural catheter placement for fear of slowing their labor or increasing the risk of caesarean section [23]. Reliable and complete information delivered by healthcare professionals might be the best solution [24]. In this work, the delegation of such information to anesthetic nurses made it possible to dedicate more time to women, which improved both parturient and anesthetist satisfaction; while duration of the anesthesia consultation and recourse of expectant mothers to other sources of information were reduced. This session was based on a multimodal approach to information combining computer presentation, oral discussion, presentation of material and a written leaflet to take home, which helped to reach a maximum of participants. Thus, improving knowledge and better understanding the benefit-risk balance of epidural analgesia, probably helped women better manage the analgesia provided for their labor and placed them at the center of the decision-making process, which has been recognized as improving satisfaction with childbirth [25–27]. In addition, information received from nurses decreased women’s anxiety before epidural catheter placement, which may have contributed to increased satisfaction [28]. Finally, having been informed by anesthetic nurses was the only factor that positively impacted satisfaction after adjustment, overriding the influences of age, socioeconomic status, or occurrence of complications during delivery when new mothers evaluated their childbirth experience.

Our study present limitations. First, the clinical significance of a 1-point increase in the satisfaction score may be discussed. While it appears modest at first glance, it should be emphasized that satisfaction in the conventional group was already high (9/10 and 8/10 for information and experience of delivery, respectively), which makes it more difficult to detect an important effect related to the new information modality. It could be hypothesized that the magnitude of this effect may be even higher in other centers in which satisfaction with information received is currently lower. However, beyond this slight increase of maternal satisfaction, the intervention was significantly associated with other positive endpoints for new mothers (improvement of knowledge, decreased anxiety), but also for anesthetists, anesthetic nurses, and even obstetricians and midwives. This was evidenced by the unpleasant feeling of going backwards expressed by all the staff of our maternity, when these information sessions were interrupted because of the first COVID-19 epidemic wave. We acknowledge that the design of our study does not allow determining which component of our intervention (i.e. delegation to anesthetic nurses, diversification of presentation media, and extension of information time) had the most beneficial impact. Nevertheless, we believe that improving new mothers’ satisfaction with their childbirth thanks to better information regarding a stressful procedure, is a relevant goal to achieve whatever its magnitude, notably as bad experience of childbirth has been identified as a risk factor for developing post-partum psychological disorders [29, 30].

Second, our study was non-randomized. However, randomization of parturient women would have generated organizational difficulties. Indeed, the information session must take place before the anesthesia consultation, so that the anesthetist can individually validate epidural analgesia indication with women. Consequently, randomization should have been done prior to the consultation, whereas it represents the first contact of parturient women with the anesthetic team during uncomplicated pregnancy in French organization. In addition, information about the two arms before patient’s consent would have introduced a bias and probably high rate of refusal or consent withdrawal as expectant mothers randomized in the “conventional information” group would have been informed of the possibility of receiving extended multimodal information. Thus, we preferred conducting a prospective sequential study with the precaution of including a number of women based on an *a priori* sample size calculation, and in a short period of time to minimize the risk of any other practice changes between the two periods.

Finally, we acknowledge that the exclusion of women who gave birth without epidural analgesia, imposed by organizational constraints and limited resources that made it difficult to screen daily all parturient women admitted for unplanned deliveries to identify women included in the study, may have introduced a selection bias.

In conclusion, an extended multimodal information session on epidural analgesia delegated to anesthetic nurses is associated with improved parturient women’s satisfaction, while durably increasing their knowledge and reducing their anxiety. Such information modality may be incorporated into maternity wards to improve new mothers’ childbirth experience.

## Supplementary Information

**Additional file 1.** STROBE Statement—checklist of items that should be included in reports of observational studies

**Additional file 2.** 

## Data Availability

The datasets generated and analyzed during the current study are not publicly available due to preservation of the individual’s privacy under the European General Data Protection Regulation but are available from the corresponding author on reasonable request.
