# Supplementary material for: Epidural analgesia information sessions provided by anesthetic nurses: impact on satisfaction and anxiety of parturient women a prospective sequential study
Source: BMC Anesthesiol. 2022 Apr 12;22:105. doi: 10.1186/s12871-022-01647-z (PMC9002221; doi:10.1186/s12871-022-01647-z)
Supplement: Supplementary file 2 — Additional file 2. [file 12871_2022_1647_MOESM2_ESM.docx]

**Impact of dedicated information on epidural analgesia for labour made by anaesthetic nurses on satisfaction and anxiety of parturient women: a prospective sequential study**

**SUPPLEMENTARY MATERIAL: knowledge test on epidural analgesia for parturient women**

**First question** *(one or several correct answers)*

Epidural for labor and delivery:

- 1. is used to provide general anaesthesia
  2. aims to stop feeling the contractions
  3. helps to reduce the pain due to contractions and delivery
  4. allows a continuous infusion of analgesic medication
  5. prevents moving your legs

**Second question** *(one or several correct answers)*

Epidural may be placed by:

1. midwifes
2. anaesthetist residents
3. anaesthetist physicians
4. anaesthetist nurses
5. obstetric physicians

**Third question** *(one or several correct answers)*

Epidural may:

1. be more effective on the right side than the left side of the body
2. cause headaches after the delivery
3. cause a drop in blood pressure
4. increase the risk of having a caesarean section
5. anaesthetise the baby

**Fourth question** *(one or several correct answers)*

An epidural for your delivery:

1. allows you to drink water all along the labour and delivery
2. allows performing a caesarean section without having a general anaesthesia
3. allows you to breastfeed your baby after the delivery
4. allows you to walk immediately after the delivery
5. cannot be performed if you suffer from chronic back pain
